# Increased Prevalence of Human Polyomavirus JC Viruria in Chronic Inflammatory Rheumatic Diseases Patients in Treatment with Anti-TNF α: A 18 Month Follow-Up Study

**DOI:** 10.3389/fmicb.2016.00672

**Published:** 2016-05-10

**Authors:** Donatella Maria Rodio, Elena Anzivino, Monica Mischitelli, Anna Bellizzi, Rossana Scrivo, Daniela Scribano, Gianlorenzo Conte, Carla Prezioso, Maria Trancassini, Guido Valesini, Anna Teresa Palamara, Valeria Pietropaolo

**Affiliations:** ^1^Department of Public Health and Infectious Diseases, “Sapienza” University of RomeRome, Italy; ^2^Department of Public Health and Infectious Diseases, Institute Pasteur, Cenci-Bolognetti Foundation, “Sapienza” University of RomeRome, Italy; ^3^Department of Internal Medicine and Medical Disciplines, Rheumatology, “Sapienza” University of RomeRome, Italy; ^4^Department of Experimental and Clinical Sciences, “G. D'Annunzio” University of ChietiChieti, Italy; ^5^San Raffaele Pisana Scientific Institute for Research, Hospitalization and Health CareRome, Italy; ^6^Sbarro Institute for Cancer Research and Molecular Medicine, Center for Biotechnology, College of Science and Technology, Temple UniversityPhiladelphia, PA, USA

**Keywords:** human polyomavirus JC, chronic inflammatory rheumatic diseases, anti-TNF-α, NCCR, VP1

## Abstract

Chronic inflammatory rheumatic diseases (CIRDs) are immune-mediated pathologies involving joints. To date, TNFα-blocking agents administration is the most promising therapy, although these treatments are associated with an increased Polyomavirus JC (JCPyV) reactivation, the etiological agent of the Progressive Multifocal Leukoencephalopathy (PML). The aim of this study was the recruitment and the analysis of a CIRDs cohort in order to investigate a possible correlation between JCPyV presence and the influence of anti-TNF-α agents on viral loads. Blood and urine samples were collected from 34 CIRDs subjects prior the first anti-TNF-α infusion (T0) and after 3 (T3), 6 (T6), 12 (T12), and 18 (T18) months. Results showed persistent JC viruria significantly higher than JC viremia throughout the 18 month follow-up study (*p* = 0.002). In JCPyV positive samples, the non-coding control region (NCCR) was analyzed. Results evidenced archetypal structures (type II-S) in all isolates with the exception of a sequence isolated from a plasma sample, that corresponds to the type II-R found in PML subjects. Finally, the viral protein 1 (VP1) genotyping was performed and results showed the prevalence of the European genotypes 1A, 1B, and 4. Since only few studies have been carried out to understand whether there is a PML risk in CIRDs population infected by JCPyV, this study contributes to enrich literature insight on JCPyV biology in this cluster. Further investigations are necessary in order to recognize the real impact of biologics on JCPyV life cycle and to identify possible and specific viral variants related to increased virulence in CIRDs patients.

## Introduction

Chronic Inflammatory Rheumatic Diseases (CIRDs) are severe and chronic pathologies that cause deterioration of the quality of life and place a major burden on health care systems worldwide (Mócsai et al., 2014).

Rheumatic Arthritis (RA) is a multiple joints inflammatory disease associated with an increased mortality when autoantibodies can be detected in the serum (Gerlag et al., 2015; Lahiri and Dixon, 2015). Its prevalence rate is approximately 1%, peaking between the ages of 35 and 50 years (Kelly, 2015).

Ankylosing spondylitis (AS) is a chronic inflammatory disease that affects 1% of the general population. Pathogenesis causes destruction and fusion of the spinal vertebrae and sacroiliac joints and the ligament calcification process, which results in pain (Ghasemi-Rad et al., 2015).

Psoriatic arthritis (PsA) is a systemic inflammatory arthritis characterized by chronic and progressive disease leading to lost productivity and reduced quality of life. PsA affects men and women equally (Dewing, 2015). PsA is a distinct disease respect to RA and AS, nevertheless, disease-modifying anti-rheumatic drugs (DMARDs) were introduced into the therapy of all these inflammatory rheumatic diseases to provide symptomatic improvement and to progress or maintain quality of life (Moll and Wright, 1973; Bijlsma, 2012). Methotrexate (MTX) is the DMARD most widely used and it remains the first line therapy nevertheless its cytotoxic nature and/or partial efficacy limit its use (Mócsai et al., 2014).

Pro-inflammatory cytokines are also involved in the pathogenesis of many inflammatory and autoimmune diseases since their overexpression. Therefore, to counteract this phenomenon, synthetic glucocorticoids (GCs) are widely used, unfortunately, many patients show unresponsiveness called GC resistance (GCR) (Dejager et al., 2014).

Therapeutics molecules of biologic origin are named biologic agents, among them, drugs that block tumor necrosis factor alpha (TNF-α) were introduced into the therapy of autoimmune and inflammatory diseases in the late 1990s (Mócsai et al., 2014). Unfortunately, treatments with biologicals are increasingly associated with amplified susceptibility to viral infections, in particular, new studies are performed to understand the role of Polyomavirus John Cunningham (JCPyV) in development of a demyelinating disease, named Progressive Multifocal Leukoencephalopathy (PML), in patients treated with anti-TNF α (Comar et al., 2013). PML occurs as a consequence of lytic infection of oligodendrocytes with a tumultuous disease course and poor prognosis within a few months. It is still unclear whether the virus seeds into the Central Nervous System (CNS) during primary viremia or following latency escape (secondary viremia), in fact, whether JCPyV is carried into the brain by infected lymphocyte or whether it directly crosses the brain barrier, is a matter of debate (Hirsch et al., 2013). JCPyV is a non-enveloped icosahedron constituted by three viral proteins (VP1-3) with VP1 being the major constituent and determines receptor specificity and viral genotypes (Agostini et al., 2001; Ferenczy et al., 2012).

There are several distinct PML risk populations. The largest is the human immunodeficiency virus positive PML population. the second one is represented by patients with various forms of hematological malignancies and at present, the third-largest risk population is relapsing–remitting multiple sclerosis patients treated with natalizumab. Anti-JCV antibody positive patients are the main at risk and present a survival rate of 77% based on 517 PML patients with average follow up of almost 3 years after PML diagnosis. However, 40% of survivors had severe disability, 47% had moderate disability and 13% had mild disability (Pavlovic et al., 2015).

Because of the low PML incidence reported in patients with RA, the potential positive predictive value of the anti-JCV assay would be too low to serve as a valuable risk-mitigation option. If patients with RA are risk stratified assuming an anti-JCV antibody seropositivity of 60%, theoretically 23,400 anti-JCV antibody-positive patients would have to receive rituximab before potentially observing 1 PML case. Cases of rituximab-associated PML have often been characterized by underlying diagnoses and prior immunosuppressant use (Borie and Kremer, 2015).

Poor data are present in literature regarding the possible link between administration of etanercept and JCV infection or reactivation in treated patients. However, in 2012, Nardis et al., found JCV DNA in 2/15 PsA patients and in the same year, in a 23-year-old Native American woman with a history of systemic lupus erythematosus and erosive polyarthritis treated with prednisone and etanercept, PML was diagnosed (Graff-Radford et al., 2012; Nardis et al., 2012).

Infliximab is used for the treatment of Crohn's disease (CD), severe forms of plaque psoriasis, RA and spondyloarthritis hence, its association with JCV infection/reactivation in treated patients is largely studied. In an observational study published in 2013 by Bellizzi et al., JCV DNA was found in biological samples of either CIRDs and CD patients treated with this medication. In 2 CD subjects a rearranged form of the viral non-coding control region (NCCR) was also found in colon-rectal biopsies after 16 months of therapy (Bellizzi et al., 2013a).

NCCR is hyper-variable, contains a promoter/enhancer arrangement responsible for neurotropism and neuro-virulence affecting viral transcription and replication (Ferenczy et al., 2012). Four distinct structural forms of NCCR (I-S, I-R, II-S, and II-R) are defined along with tissue tropisms: type II-S is identified as archetype CY and it is composed of six boxes named A-F. Each box contains binding sites for transcriptional cell factors involved in viral transcription. These binding sites undergo to deletion and enhancement process generating variants that could up-modulate viral expression in a specific anatomical site and could alter the cellular host range (Jensen and Major, 2001; Tan and Koralnik, 2010). Type I-S is 98 bp long and it is composed of boxes A, C and E. Type I-R has repeats of this 98 bp unit, with various deletions, as seen in the JCPyV prototype Mad-1 and Mad-4 strains; both of these types have no boxes B and D (Jensen and Major, 2001; Tan and Koralnik, 2010). Mad-1 strain was isolated from tissues of PML patients (Jensen and Major, 2001; Tan and Koralnik, 2010) and it was named on the hypothesis that it results from a rearrangement of the archetype (Marshall and Major, 2010).

Finally, type II-R is composed by *rearranged NCCRs* characterized by repeats of the 98 bp unit with inserts and various mutations (Jensen and Major, 2001; Tan and Koralnik, 2010). Rearranged NCCRs can correlate with PML poor clinical outcome (Marshall and Major, 2010).

Although viral infection and replication occurs at very high incidence, as demonstrated by Egli et al., on 400 consecutive blood donors (Egli et al., 2009), PML is a rare disease, nevertheless, its onset as a side effect of immune-modulatory therapy, is a growing concern with reports of fatal cases (Molloy and Calabrese, 2012; Bellizzi et al., 2013b). Therefore, continued research and a more detailed understanding of JCPyV biology, epidemiology and pathology are of increasing importance.

On these bases, the objective of this study was to recruit and to analyze a cohort of subjects affected by CIRDs, including RA, AS and PsA, in order to evaluate JCPyV circulation in CIRDs patients and the possible influence of anti-TNF-α agents on viral loads. In addition, in JCPyV positive samples, NCCR was analyzed to perceive whether particular rearrangements could have arisen in a specific CIRD. Finally, in order to identify the most prevalent JCPyV genotype in CIRDs cohort, VP1 genotyping was performed.

## Materials and methods

### Patients and samples

In this study, 34 subjects affected by CIRDs (12 males, 22 females) were dynamically enrolled. Patient's mean age ± standard deviation (std. dev.) was 52 ± 12 years (range: 25–74 years) and the mean disease duration was 115.4 ± 116.1 months (range: 9–480 months). Among these 34 outpatients, 8 were affected by RA (8 females), 8 were affected by AS (4 males, 4 females) and 18 patients were affected by PsA (10 males, 8 females). This cohort, referred to the Department of Internal Medicine and Medical Disciplines, Rheumatology Unit (Sapienza University of Rome, Italy), was composed by subjects enrolled from January 2013 to May 2015. Patients were treated first with DMARDs and/or GCs and successively with anti-TNF-α (golimumab, adalimumab, etanercept, certolizumab pegol), DMARDs and/or GCs when they resulted unresponsiveness exclusively to DMARDs therapy. After obtaining informed consent, basic demographic data including concomitant medications and disease activity measures, were collected and recorded on a standardized form, just before the beginning of biologic therapy. All patients were classified according to standard criteria (Van der Linden et al., 1984; Arnett et al., 1988; Taylor et al., 2006), designated to start biologic treatment and consecutively enrolled. Each patient was evaluated by the same rheumatologist. RA and PsA clinical evaluation included: swollen (SJC) and tender joint count (TJC), patient and physician global assessment on a visual analog scale (VAS, 0–100 mm), and HAQ (Fries et al., 1982). The HAQ scores range from 0 to 3, with a higher score indicating a higher level of disability (Lesuis et al., 2012). A blood drawing was also performed to evaluate ESR (mm/h) and CRP (mg/l). Disease activity was assessed by the DAS28-CRP and/or the DAS28-ESR, with a high score indicating more active disease (Lesuis et al., 2012). Current disease activity in patients with AS was measured by the Bath Ankylosing Spondylitis Disease Activity Index (BASDAI), ranging from 0 (no activity) to 10 (maximum activity) (Garrett et al., 1994) (Tables 1, 2).

**Table 1:** **Clinical data of patients affected by CIRDs at baseline and during follow-up**.

**Parameters**	**T0**	**T3**	**T6**	**T12**	**T18**	***p*-value^§^**
M/F (n) n patients	12/22 34	12/21 33	11/21 32	10/22 32	11/21 32	
Age° (median/range)	52/25–74					
Diagnosis: PsA/AR/AS	18/8/8	17/8/8	17/8/7	17/8/7	16/8/8	
Months of disease (median/range)	66/9–480					
CRP (mg/dl; median/25th-75th percentile)	0.22/0.08–0.75	0.19/0.08–0.53	0.15/0.09–0.49	0.23/0.14–0.77	0.31/0.16–0.56	0.416
ESR (mm/h; median/25th–75th percentile)	14/8–25	13/6–20	14/7–19	11/5–21	16/6–19.3	0.580
HAQ (0–3; mean/SD)	0.92 ± 0.72	0.80 ± 0.75	0.73 ± 0.72	0.70 ± 0.66	0.64 ± 0.66	0.498
DAS28-ESR (mean/SD)^*^	3.74 ± 0.97	2.69 ± 1.10	2.70 ± 0.82	2.81 ± 0.62	3.49 ± 1.08	**0.002**
DAS28-CRP (mean/SD)^*^	3.24 ± 0.85	2.60 ± 1.10	2.56 ± 0.75	2.76 ± 0.84	3.18 ± 1.27	0.085
Physician's global assessment of disease activity (0–100 mm, VAS; mean/SD)^*^	46.1 ± 19.1	26.18 ± 23.12	23.88 ± 22.61	27.03 ± 23.45	26.38 ± 20.67	<**0.0001**
Patient's global assessment of disease activity (0–100 mm, VAS; mean/SD)^*^	55.2 ± 26.6	40.15 ± 24.82	35.63 ± 25.78	34.72 ± 23.45	35.28 ± 27.83	**0.007**
BASDAI (1–10; mean/SD)^**^	5.61 ± 2.92	4.12 ± 2.82	4.58 ± 3.80	3.95 ± 2.97	4.15 ± 2.90	0.305
**BIOLOGIC AGENTS USED FOR CIRDS TREATMENT**						
*golimumab (n/%)*	10/29.4	10/30.3	10/31.3	10/31.3	10/31.3	
*Adalimumab (n/%)*	11/32.4	11/33.3	9/28.1	10/31.3	10/31.3	
*Etanercept (n/%)*	11/32.4	10/30.3	11/34.4	10/31.3	10/31.3	
*Certolizumab pegol (n/%)*	2/5.9	2/6.1	2/6.3	2/6.3	2/6.3	
Concomitant DMARDs (n/%)	10/29.4	8/24.2	8/25	7/21.9	6/18.8	
Concomitant DMARDs and glucocorticoids (n/%)	7/20.6	6/18.2	7/21.9	5/15.6	4/12.5	
Concomitant glucocorticoids (n/%)	9/26.5	3/9.1	4/12.5	4/12.5	8/25	
No concomitant treatment (n/%)	4/11.8	15/45.5	10/31.5	11/34.4	12/37.5	
Other concomitant treatments (n/%)	4/11.8	1/3.0	3/9.4	5/15.6	2/6.3	

*PsA, psoriatic arthritis; RA, rheumatoid arthritis; AS, ankylosing spondylitis; CRP, C-reactive protein; ESR, erythrocyte sedimentation rate; HAQ, Health Assessment Questionnaire; SD, standard deviation; DAS28, 28-joint Disease Activity Score; VAS, visual analog scale; BASDAI, Bath Ankylosing Spondylitis Diseease Activity Index; DMARDs, disease modifying anti-rheumatic drugs*.

* *For patients with RA and PsA*.

** *For patients with AS and PsA. °Age: expressed in years*.

§ *By Kruskal-Wallis test. p < 0.05 was considered statistically significant. Bold values are statistically significant*.

**Table 2:** **Clinical data of patients affected by RA, AS and PsA at baseline (T0)**.

**Diagnosis**	***PsA***	***RA***	***AS***	***p*-value^§^**
Total	18	8	8	
M/F (n)	8/10	0/8	4/4	
Age° (median/range)	51.5/38–72	64/40–74	41/25–59	
Months of disease (median/range)	72/24–480	96/24–360	48/9–168	
CRP (mg/dl; median/25th–75th percentile)	0.20/0.08–0.44	0.5/0.05–0.95	0.80/0.44–2.86	0.087
ESR (mm/h; median/25th–75th percentile)	13.5/8.8–20.8	20.5/13.3–39	9.0/7.0–28.5	0.506
HAQ (0-3; mean/SD)	0.87 ± 0.73	1.28 ± 0.89	0.80 ± 0.57	0.344
DAS28-ESR (mean/SD)^*^	3.60 ± 0.97	4.27 ± 1.01	/	0.193
DAS28-CRP (mean/SD)^*^	3.20 ± 0.85	3.60 ± 1.00	/	0.350
Physician's global assessment of disease activity (0–100 mm, VAS; mean/SD)^*^	43.72 ± 21.28	44.75 ± 19.65	53.88 ± 12.06	0.147
Patient's global assessment of disease activity (0–100 mm, VAS; mean/SD)^*^	49.67 ± 27.08	60.75 ± 27.20	64.25 ± 24.42	0.348
BASDAI (1–10; mean/SD)^**^	5.39 ± 2.48	/	6.23 ± 3.66	0.624
**Biologic agents used for treatment**				
*Golimumab (n/%)*	5/27.8	/	5/62.5	
*Adalimumab (n/%)*	5/27.8	3/37.5	3/37.5	
*Etanercept (n/%)*	8/44.4	3/37.5	/	
*Certolizumab pegol (n/%)*	/	2/25	/	
Concomitant DMARDs (n/%)	5/27.8	4/50	1/12.5	
Concomitant DMARDs and glucocorticoids (n/%)	3/16.7	3/37.5	1/12.5	
Concomitant glucocorticoids (n/%)	6/33.3	1/12.5	2/25	
No concomitant treatment (n/%)	3/16.7	/	1/12.5	
Other concomitant treatments (n/%)	1/5.6	/	3/37.5	

*PsA, psoriatic arthritis; RA, rheumatoid arthritis; AS, ankylosing spondylitis; CRP, C-reactive protein; ESR, erythrocyte sedimentation rate; HAQ, Health Assessment Questionnaire; SD, standard deviation; DAS28, 28-joint Disease Activity Score; VAS, visual analog scale; BASDAI, Bath Ankylosing Spondylitis Diseease Activity Index; DMARDs, disease modifying anti-rheumatic drugs*.

* *For patients with RA and PsA*.

** *For patients with AS and PsA. °Age: expressed in years*.

§ *By Kruskal-Wallis test or by Mann–Whitney U-test. p < 0.05 was considered statistically significant*.

From each of these 34 outpatients, a sample of urine, plasma and peripheral blood mononuclear cells (PBMCs) was collected prior anti-TNF-α agents treatment (baseline designated as t0) and during the follow-up at 3 (T3), 6 (T6), 12 (T12), and 18 months (T18) after starting biological therapy. In particular, 33 urine and blood samples were collected from 33 patients at T3, 31 urine and 32 blood samples were collected from 32 patients at T6, 31 urine and 32 blood samples were collected from 32 patients at T12 and finally 32 urine and blood samples were collected from 32 patients at T18.

### Clinical specimens processing and JCV DNA extraction

DNA for molecular analysis was extracted from 500 μl of each urine, collected without preservatives, using the DNeasy® Blood & Tissue Kit (QIAGEN, S.p.A, Italy) according to the manufacturer's instructions. Blood samples, collected in 4-mL Vacutainer® tubes containing EDTA (BD Becton Dickinson S.p.A, Italia), were centrifuged at 1.376 g/s for 10 min and DNA was extracted from 200 μL of plasma using the DNeasy® Blood & Tissue Kit (QIAGEN, S.p.A, Milan, Italy). PBMCs were isolated from whole blood using the standard Ficoll Hypaque density gradient centrifugation technique (Bøyum et al., 1991), and the number of viable leukocytes was determined by trypan blue exclusion. PBMCs DNA extraction was performed on 10^6^ cells by the QIAmp® DNA Blood Kit (QIAGEN S.p.A, Milan, Italy) according to the manufacturer's instruction. DNA yield of all biological specimens was determined by measuring its concentration in the eluate by absorbance at 260 nm and then stored at −20°C until use.

Standard laboratory procedures for sterile DNA extraction and PCR were practiced for all specimens.

### Quantitative PCR (Q-Pcr) for JCPyV TAg

DNA extracted from each sample was tested for JCV genome detection and quantification by Q-PCR following a published protocol (Delbue et al., 2008). Each sample was analyzed in duplicate and the viral loads were given as the mean of at least two positive reactions. Standard precautions designed to prevent contamination were followed and a negative control was included in each run. Viral DNA was quantified using a standard curve consisted of serial dilutions at known titer of a plasmid containing the entire JCPyV genome. For urine and plasma, viral DNA was expressed as genome equivalents (gEq)/ml and as genome equivalents (gEq)/10^6^ cells for PBMCs.

### PCR for JCPyV NCCR

In order to amplify the JCPyV NCCR, a nested-PCR employed two pairs of primers that anneal to the invariant regions flanking JCPyV NCCR was performed (Pietropaolo et al., 2003). The first pair primers amplified a 724 bp DNA fragment in JCPyV (Mad-1) whereas the second pair annealed to a portion of the first round PCR product, generating a fragment of 308 bp (Flaegstad et al., 1991; Markowitz et al., 1993). Standard precautions designed to prevent contamination were followed and a negative control was included in each run (Kwok and Higuchi, 1989).

PCR products were detected by electrophoresis on a ethidium bromide-stained 2% agarose gel and visualized under UV light.

### PCR for JCPyV VP1

In order to define the JCPyV genotype of the isolated viral strains, a 215-bp fragment of the JCPyV VP1 gene was amplified using a single set of primers (Agostini et al., 2001).

PCR products were detected by electrophoresis on a 2% agarose gel stained with ethidium bromide and visualized under UV light.

### Sequencing of JCPyV NCCR and VP1 regions

PCR products corresponding to JCPyV NCCR and VP1 regions were purified prior to sequencing (Pietropaolo et al., 2003). DNA sequencing was performed in service (BioFab research s.r.l., Rome, Italy). All sequences obtained from NCCR amplicons were compared to JCPyV NCCR of the prototype Mad-1 (GenBank: J02227) and of the archetype CY (GenBank: AB038249.1). Sequences corresponding to VP1 amplicons, were classified into the known genotypes/subtypes according to the single nucleotide polymorphisms (SNPs) patterns and aligned with those reported by Jobes et al. (2001). Sequence alignments were performed with ClustalW2 at the EMBL-EBI website using default parameters (ClustalW2 - multiple sequence alignment^1^).

### Cloning and sequencing

PCR amplicons corresponding to the exact size of JCPyV NCCR, were cloned into a pGEM-T Easy Vector (Promega Corporation, Madison, WI). Plasmid DNA was purified from up to 25 clones of amplicons, visualized in 1% agarose gel electrophoresis and sequenced (BioFab research s.r.l., Rome, Italy). Homology between obtained sequences and/or the prototype Mad-1 and the archetype CY was searched by ClustalW2 at the EMBL-EBI website (ClustalW2 - multiple sequence alignment).

### Data analysis

Data were summarized as medians and ranges or as mean ± standard deviation, as appropriate. If *Z*-test indicated a non-normal distribution, we used non-parametric tests such as Mann–Whitney *U*-test and Kruskal–Wallis test. Categorical data were analyzed by using χ^2^-test and Student's *t*-test. *P* < 0.05 were considered statistically significant.

## Results

### Follow-up of JC viral load in urine, plasma and PBMCs samples collected from CIRDs patients

In this study, 34 subjects affected by CIRDs (12 males, 22 females) were dynamically enrolled. These outpatients were divided in three different classes on the basis of their pathology: 8 RA (8 females), 8 AS (4 males, 4 females) and 18 PsA (8 males, 10 females). Patients demographic and clinical characteristics are shown in Tables 1, 2. None of the patients developed neurological symptoms suggestive of PML at any time point of this 18 month follow-up study.

**Figure 1:**
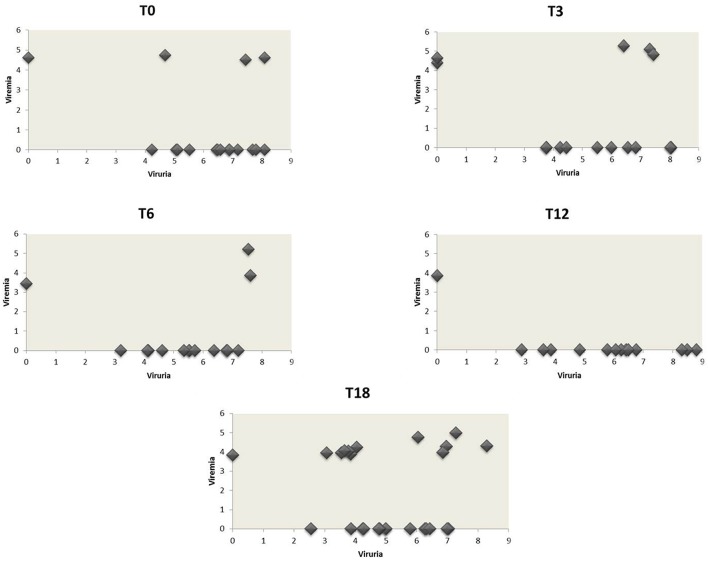
**JCPyV urine loads vs. the corresponding plasma viral loads in CIRDs patients during the 18 month follow-up study**. The values of JCPyV viruria for each patient at the different time-points were reported on the x-axis whereas the corresponding viremia values on the y-axis. All JCPyV loads values were expressed as median of log10 genome equivalent (gEq)/mL in urine and in plasma. Viremia was mainly associated to viruria during the entire follow-up.

At baseline, 34 urine were screened for the presence of JCPyV DNA. Q-PCR revealed that 16/34 (47.1%) urine were JCPyV-positive with a median value of 6.73 log10 (gEq)/ml (range: 4.23–8.10) (Tables 3, 4). The median values of each class are reported in Tables 3, 4. Regarding the 34 plasma samples collected at baseline, 4/34 (11.8%) patients (1 RA, 1 AS and 2 PsA) were found positive to JCPyV DNA with a median value of 4.62 log10 (gEq)/ml (range: 4.51–4.74) (Tables 3, 4). The median values of each class are shown in Table 4. Moreover, 3 out of these 4 patients showed also JC viruria (Figure 1). Finally, PBMCs samples were all negative for JCV DNA (Tables 3, 4).

**Table 3:** **JC viral load in biological specimens collected from patients with Chronic Inflammatory Rheumatic Diseases at baseline and during the follow-up**.

	**Pt (n)^a^**	**Pt JCPyV + (n)^b^**	**Pt JCPyV − (n)^b^**	**Urine**		**Plasma**		**PBMCs**		***p*-value^e^**
				**JCPyV+/JCPyV −(NA)^c^**	**log10 gEq/mL (range)^d^**	**JCPyV+/JCPyV − (NA)**	**log10 gEq/mL (range)^d^**	**JCPyV+/JCPyV − (NA)**	**log10 gEq/10^6^c (range)^d^**	
T0	34	17	17	16 ÷ 18	6.73 (4.23–8.10)	4 ÷ 30	4.62 (4.51–4.74)	0 ÷ 34	/	0.011
T3	33 (1)	16	17	13 ÷ 20 (1)	6.41 (3.74–8.04)	5 ÷ 28 (1)	4.83 (4.41–5.28)	3 ÷ 30 (1)	3.38 (2.81–3.76)	0.183
T6	32 (2)	15	17	14 ÷ 17 (3)	6.05 (3.20–7.61)	3 ÷ 29 (2)	3.87 (3.45–5.20)	0 ÷ 32 (2)	/	0.059
T12	32 (2)	11	21	13 ÷ 18 (3)	6.24 (2.87–8.80)	1 ÷ 31 (2)	3.86	2 ÷ 30 (2)	3.37 (3.39–3.35)	0.321
T18	32 (2)	27	5	25 ÷ 7 (2)	4.99 (2.55–8.29)	12 ÷ 20 (2)	4.05 (3.83–4.98)	21 ÷ 11 (2)	3.10 (2.06–3.77)	0.098
*p*-value^e^					0.121		0.045		0.603	
RR					2.08					
(IC 95%)					(1.70–2.55)					
*p*-value^f^					0.00001					

a *Pt, patients; n, number of patients*.

b *Pt JCPyV+ and Pt JCPyV−, number of patients with or without JCPyV DNA in at least 1 sample of plasma and/or PBMCs and/or urine*.

c *NA, sample not available*.

d *JCV loads values were expressed as median (range) of log10 genome equivalent (gEq)/mL in urine and in plasma, and as median (range) log10 gEq/10^6^ cells in PBMCs (gEq/10^6^ c)*.

e *By Mann–Whitney U-test and by Kruskal-Wallis test. p < 0.05 was considered statistically significant*.

f *Relative risk (RR) and 95% confidence interval (95% CI) statistically significant with a p < 0.05 by χ2-test*.

**Table 4:** **JC viral load in biological samples collected from patients with psoriatic arthritis, rheumatoid arthritis and ankylosing spondylitis at baseline and during the follow-up**.

**Sampling time**		**CIRDs**	***PsA***	***RA***	***AS***	***p*-valu*e***
T0^*^	Plasma JCPyV+/JCPyV−	4 ÷ 30	2 ÷ 16	1 ÷ 7	1 ÷ 7	0.079
	Plasma log10 JCPyV load (range)^§^	4.62 (4.51–4.74)	4.57 (4.51−4.62)	4.74	4.62	
	25th–75th percentile	4.59–4.65	/	/	/	
	Urine JCPyV+/JCPyV−	16 ÷ 18	7 ÷ 11	5 ÷ 3	4 ÷ 4	
	Urine log10 JCPyV load (range)^§^	6.73 (4.23–8.10)	6.48 (4.23–8.09)	5.11 (4.69–6.89)	7.74 (6.58–8.10)	
	25th–75th percentile	5.42–7.51	5.99–7.31	5.07–6.88	7.41–7.88	
	PBMCs JCPyV+/JCPyV−	0 ÷ 34	0 ÷ 34	0 ÷ 34	0 ÷ 34	
	PBMCs log10 JCPyV load (range)^§^	/	/	/	/	
T3^*^	Plasma JCPyV+/JCPyV−	5 ÷ 28 (1NA)	4 ÷ 13 (1NA)	1 ÷ 7	0 ÷ 8	0.942
	Plasma log10 JCPyV load (range)^§^	4.83 (4.41–5.28)	4.75 (4.41–5.28)	5.10	/	
	25th–75th percentile	4.66–5.10	4.60–4.94	/	/	
	Urine JCPyV+/JCPyV−	13 ÷ 20 (1NA)	4÷13 (1NA)	4 ÷ 4	5 ÷ 3	
	Urine log10 JCPyV load (range)^§^	6.41 (3.74–8.04)	6.62 (4.23–7.43)	6.27 (4.44–7.30)	5.50 (3.74–8.04)	
	25th–75th percentile	4.44–7.30	5.87–6.97	5.60–6.74	3.76–8.01	
	PBMCs JCPyV+/JCPyV−	3 ÷ 30 (1NA)	1 ÷ 16 (1NA)	1 ÷ 7	1 ÷ 7	
	PBMCs log10 JCPyV load (range)^§^	3.38 (2.81–3.76)	2.81	3.38	3.76	
	25th–75th percentile	3.10–3.57	/	/	/	
T6^*^	Plasma JCPyV+/JCPyV−	3 ÷ 29 (2NA)	1 ÷ 16 (1NA)	1÷7	1÷6 (1NA)	0.424
	Plasma log10 JCPyV load (range)^§^	3.87 (3.45–5.20)	3.45	5.20	3.87	
	25th–75th percentile	3.66–4.54	/	/	/	
	Urine JCPyV+/JCPyV−	14 ÷ 17 (3NA)	6 ÷ 10 (2NA)	5 ÷ 3	3 ÷ 4 (1NA)	
	Urine log10 JCPyV load (range)^§^	6.05 (3.20–7.61)	6.45 (3.20–6.82)	5.73 (4.11–7.53)	7.19 (4.61–7.61)	
	25th–75th percentile	4.80–6.82	5.73–6.73	4.14–5.72	5.90–7.40	
	PBMCs JCPyV+/JCPyV−	0 ÷ 32 (2NA)	0 ÷ 17 (1NA)	0 ÷ 8	0 ÷ 7 (1NA)	
	PBMCs log10 JCPyV load (range)^§^	*/*	/	/	/	
T12^*^	Plasma JCPyV+/JCPyV−	1 ÷ 31 (2NA)	1 ÷ 16 (1NA)	0 ÷ 8	0 ÷ 7 (1NA)	0.821
	Plasma log10 JCPyV load (range)^§^	3.86	3.86	/	/	
	Urine JCPyV+/JCPyV−	13 ÷ 18 (3NA)	6 ÷ 11 (1NA)	4 ÷ 3 (1NA)	3 ÷ 4 (1NA)	
	Urine log10 JCPyV load (range)^§^	6.24 (2.87–8.80)	6.10 (3.60–8.80)	6.41 (4.84–8.48)	6.24 (2.87–8.30)	
	25th–75th percentile	4.84–6.76	4.34–6.47	5.76–7.19	4.56–7.27	
	PBMCs JCPyV+/JCPyV−	2 ÷ 30 (2NA)	1 ÷ 16 (1NA)	0 ÷ 8	1 ÷ 6 (1NA)	
	PBMCs log10 JCPyV load (range)^§^	3.37 (3.35–3.39)	3.39	/	3.35	
T18^*^	Plasma JCPyV+/JCPyV−	12 ÷ 20 (2NA)	6÷10 (2NA)	3 ÷ 5	3 ÷ 5	0.707
	Plasma log10 JCPyV load (range)^§^	4.05 (3.83–4.98)	4.01 (3.83–4.32)	4.24 (3.96–4.77)	4.28 (3.95–4.98)	
	25th–75th percentile	3.96–4.29	3.91–4.06	4.10–4.51	4.12–4.63	
	Urine JCPyV+/JCPyV-	25÷7 (2NA)	12÷4 (2NA)	6 ÷ 2	7 ÷ 1	
	Urine log10 JCPyV load (range)^§^	4.99 (2.55–8.29)	4.64 (2.55–8.29)	5.52 (3.06–6.99)	4.80 (3.55–7.28)	
	25th–75th percentile	3.86–6.43	3.83–6.28	4.29–6.33	4.51–7.01	
	PBMCs JCPyV+/JCPyV−	21 ÷ 11 (2NA)	10÷6 (2NA)	6 ÷ 2	5 ÷ 3	
	PBMCs log10 JCPyV load (range)^§^	3.10 (2.06–3.77)	3.05 (2.06–3.77)	3.23 (2.85–3.76)	3.21 (2.83–3.74)	
	25th–75th percentile	2.95–3.39	2.95–3.12	2.95–3.39	2.96–3.39	
	*p*-value *viruria*		0.290	0.707	0.428	

*CIRDs, chronic inflammatory rheumatic diseases; PsA, psoriatic arthritis; RA, rheumatoid arthritis; AS, ankylosing spondylitis; NA, sample not available*.

* *Times of follow up: baseline (T0) and 3, 6, 12, and 18 months (T3, T6, T12, and T18)*.

§ *JCPyV loads values were expressed as median (range) of log10 genome equivalent (gEq)/mL in urine and in plasma, and as median (range) log10 gEq/10^6^ cells in PBMCs*.

Three months after starting anti-TNF-α treatment (T3), 33 urine samples, 33 plasma and 33 PBMCs were collected (8 AR, 8 AS, and 17 PsA). Viral DNA was found in 13/33 (39.4%) urine with a median value of 5.10 log10 (gEq)/ml (range: 4.83–5.28) (Tables 3, 4). The median values of RA, AS and PsA groups are described in Table 4. About plasma, JCPyV was found in 5/33 (15.2%) samples (1 RA and 4 PsA) with a median value of 4.83 log10 (gEq)/ml (range: 4.41–5.28) (Tables 3, 4). The median values calculated for PsA and RA groups both are shown in Table 4. JCPyV was not revealed in AS patients (Table 4). Among the 5 patients with viremia, 3 were also JCV-positive in urine samples (Figure 1). Regarding PBMCs, 3 out of 33 (9.1%) patients (1 RA, 1 AS, and 1 PsA) resulted positive for JCPyV DNA with a median value of 3.38 log10 (gEq)/10^6^cells (Tables 3, 4).

At T6, corresponding to 6 months of anti-TNF-α treatment, 31 urine samples, 32 plasma and 32 PBMCs were collected from 32 patients (8 AR, 7 AS, and 17 PsA). At this time point, 14/31 (45.2%) urine were found JCPyV positive with a median value of 6.05 log10 (gEq)/ml (range: 3.20–7.61) (Tables 3, 4). The median values of each class are reported in Table 4.

Regarding plasma, 3/32 (9.4%) samples (1 RA, 1 AS, and 1PsA) were found positive to viral genome with a median value of 3.87 log10 (gEq)/ml (range: 3.45–5.20) (Tables 3, 4). The median values of the three studied classes are labeled in Table 4. 2 out of the 3 patients showed also viruria (Figure 1). Finally, no JCPyV DNA was detected in PBMCs specimens (Tables 3, 4).

After 1 year of anti-TNF-α treatment (T12), 32 patients (8 AR, 7 SA, and 17 PsA) were screened for JCPyV DNA. JCPyV viruria was found in 13 out of 31 (41.9%) specimens with a median value of 6.24 log10 (gEq)/ml (range: 2.87–8.80) (Tables 3, 4). The median values calculated for the three groups are shown in Table 4. Conversely, JCPyV viremia was detected in a single patient affected by PsA, with a viral load of 3.86 log10 (gEq)/ml (Tables 3, 4 and Figure 1). Regarding PBMCs samples, JCPyV DNA was found in two patients: one affected by AS, with a viral load value of 3.35 log10 (gEq)/ml, and the other one affected by PsA, showing a viral load of 3.39 log10 (gEq)/ml (Tables 3, 4). Both patients were also JCPyV-positive in urine samples (data not showed).

Finally, 32 urine and blood specimens, belonging to 8 AR, 8 SA, and 16 PsA, were collected 18 months after starting biologics (T18). At this time point of follow-up, JCPyV viruria was detected in 25/32 (78.1%) CIRDs patients with a median load value of 4.99 log10 (gEq)/ml (range: 2.55–8.29) (Tables 3, 4). The median values of each class are reported in Table 4. Concerning viremia, JCPyV DNA was revealed in 12 out of 32 (37.5%) CIRDs patients screened, with a median load value of 4.05 log10 (gEq)/ml (range: 3.83–4.98) (Tables 3, 4). Moreover, Table 4 showed the median values of RA, AS and PsA classes. Interestingly, all viremic patients showed viruria, with the exception of a single subject affected by PsA (Figure 1). Finally, 21/32 (65.6%) PBMC samples resulted JCPyV-positive with a median viral load of 3.10 log10 (gEq)/ml (range: 2.06–3.77) (Table 2). In particular, 6 out of these 21 PBMC samples were isolated from patients with RA, 5 from patients with AS and 10 from patients with PsA. The median JCPyV load values found are reported in Table 4. Among 21 patients with JCV-positive PBMCs, 8 showed viruria and one patient was also viremic (data not shown).

**Figure 2:**
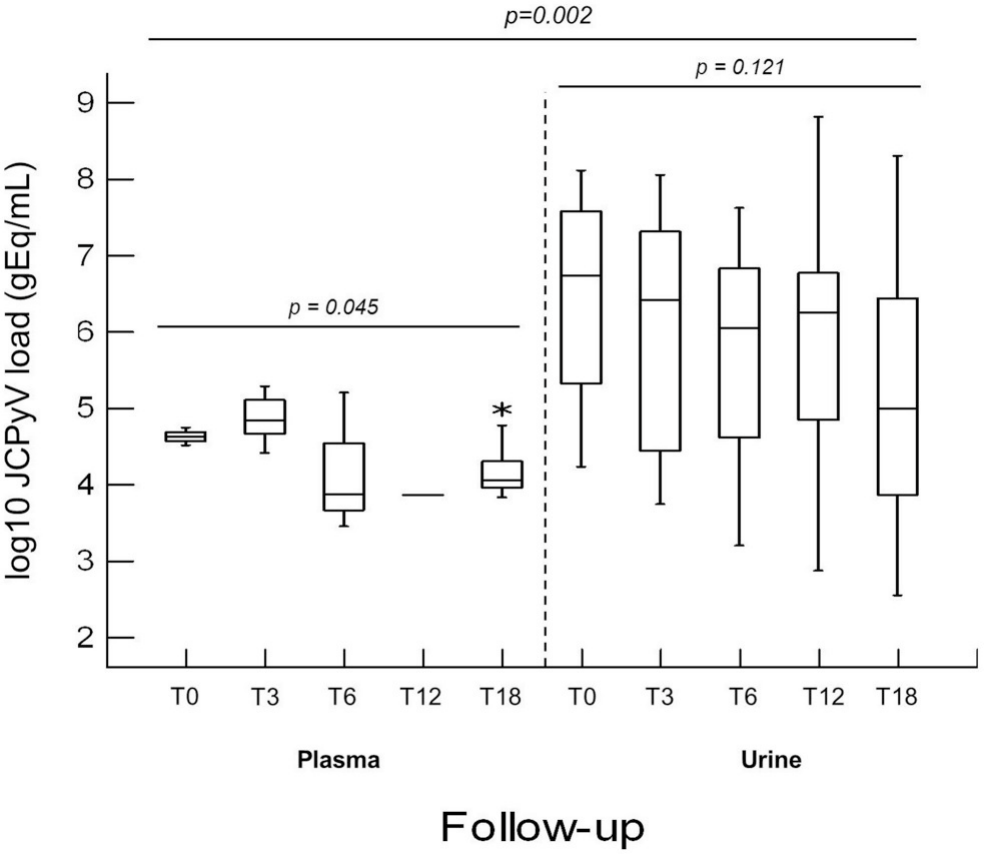
**Comparison of JCPyV viruria and viremia detected in CIRDs patients in a 18 month follow-up study**. At T0, JCPyV DNA was revealed in 47.1% urine with a median value of 6.73 log10 (gEq)/ml and in 11.8% plasma with a median value of 4.62 log10 (gEq)/ml. At T3, 39.4% of urine and 15.2% of plasma resulted JCPyV positive with a median value of 6.41 log10 (gEq)/ml and of 4.83 log10 (gEq)/ml respectively. At T6, in 45.2% urine (median value 6.05 log10 (gEq)/ml) and in 9.4% plasma (median value 3.87 log10 (gEq)/ml), JCPyV genome was found. At T12, JCPyV viruria was found in 41.9% specimens with a median value of 6.24 log10 (gEq)/ml. Conversely, JCPyV viremia was detected in a single PsA patient with a viral load of 3.86 log10 (gEq)/ml. Finally, at T18, JCPyV viruria was detected in 78.1% of CIRDs patients with a median load value of 4.99 log10 (gEq)/ml, whereas JCPyV viremia was revealed in 37.5% of samples (median value of 4.05 log10 (gEq)/ml). JCPyV viruria was significantly higher than JCPyV viremia throughout the entire follow-up (*p* = 0.002). JCPyV load values are expressed as log10 genome equivalent per milliliter (gEq/mL). T0: baseline; T3, T6, T12 and T18: 3, 6, 12, and 18 months of anti-TNF-α, therapy. ^*^indicates the highest viremia value detected in one patient.

It is noteworthy that, comparing the values of JC viruria and viremia obtained from CIRDs patients throughout the follow-up, the mean values of JC viruria were always higher than that of JC viremia (*p* = 0.002) (Figure 2). In addition, a relative risk (RR) of viral reactivation with urinary shedding equal to 2.08 (95% CI 1.70–2.55) was estimated (Table 3). Therefore, JCPyV shedding in the urine is favored respect to JCPyV circulation in the blood compartment where the virus is detected at very low incidence. However, neither the age of patients nor the administration of biologics alone nor the co-administration of DMARDs and/or GCs was different between JCPyV-positive and JCPyV-negative patients in urine (data not shown).

### PCR and sequencing analysis of JCPyV NCCR and VP1 regions

**Figure 3:**
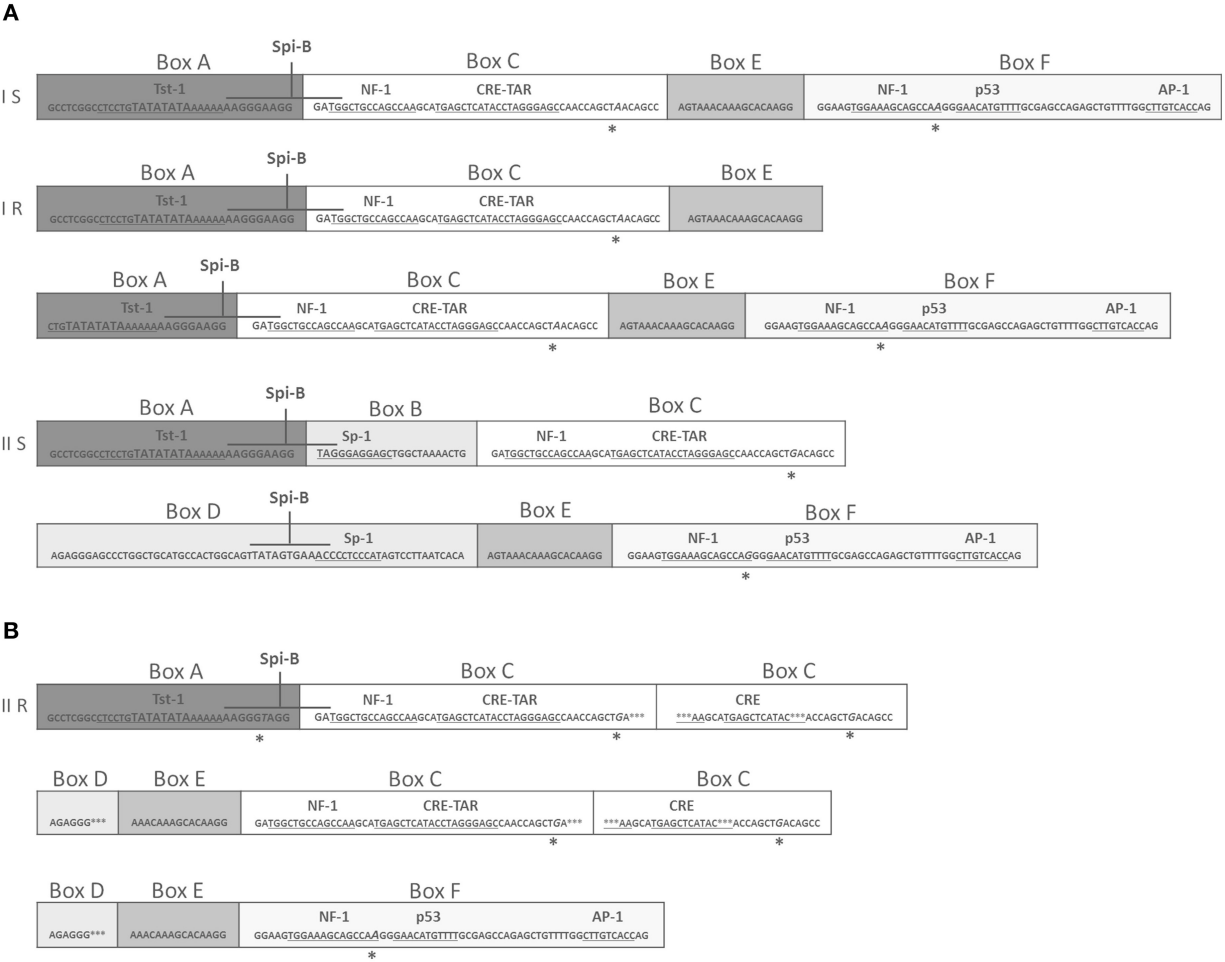
**Comparison of NCCR structural forms and rearranged NCCR II-R found in a plasma sample at T3. (A)** Type I-S is 98 base-pair (bp) long and it is composed of box A (25 bp), box C (55 bp), box E (18 bp) and F (69 bp). Type I-R has repeats of this 98 bp unit, with various deletions, as seen in the JCPyV prototype Mad-1 (GenBank no: J02227), that have no box B and box D (Jensen and Major, 2001). In particular, the prototype Mad-1 was isolated from tissues of patients with PML (Tan and Koralnik, 2010) and it was named on the hypothesis that the prototype results from a rearrangement of the archetype sequence (Comar et al., 2013). Type II-S is identified as archetype CY and it is composed of A (25 bp), B (23 bp), C (55 bp), D (66 bp), E (18 bp), and F (69 bp) boxes. It was isolated by Yogo et al. (1990). Each box contains binding sites for transcriptional cell factors involved in viral early and late transcription. These binding sites undergo to deletion and enhancement process that could generate variants that could up-modulate viral expression in a specific anatomical site (Jensen and Major, 2001). **(B)** In **(B)**, the rearranged sequence found in plasma at T3 is reported. This rearrangement presents the entire boxes A and C followed by a rearranged box C, shortened sequences of boxes D and E, a new duplication of box C (complete and rearranged), incomplete boxes D and E and finally the entire box F. Asterisks represent single nucleotide point mutations or deletions. Italicized capital letters indicate mutated nucleotides. The TATA box is presented by TATA. Boxes division from A to F is also shown. The main binding sites for transcriptional cell factors are also indicated and the corresponding nucleotides sequences are underlined. Finally, the nucleotides sequences for the transcriptional factor Spi-B are also shown in higher font.

JCPyV NCCR was searched in all Q-PCR specimens resulted positive to viral DNA. Successively, correct size amplicons were cloned, and up to 25 clones for each PCR product were sequenced to identify NCCR variants. As previously described, four distinct structural forms of JCPyV NCCR (I-S, I-R, II-S, and II-R) have been identified (Jensen and Major, 2001; Figure 3A). Results showed that all sequences belonged to type II-S (archetype CY), with the exception of a sequence isolated from a plasma sample characterized by the subsequent structure: the entire box A is directly followed by a complete box C because of the whole box B was deleted, then a rearranged box C was present and it was tailed by shortened sequence of boxes D and E. A new duplication of box C (complete and rearranged) and of incomplete boxes D and E followed by the entire box F were observed (Figure 3B).

Regarding the binding sites present in this rearranged sequence, the TATA box and the binding site for Tst-1, required for JCPyV replication and transcription of early and late genes, respectively, were both well conserved within the box A (Figure 3B). About the multiple duplication of box C, it enhances the binding site for the cyclic AMP (cAMP) response element (CRE), a protein that up-modulates JCPyV expression in cells, and for NF-1, responsible for the JCPyV neurotropism (Figure 3B). In addition, the binding sites for the cellular factors NF-1, p53, and AP-1, involved in early viral transcription and in neurotropism of the virus, are present within the box F, although a single point mutation occurred into the NF-1 binding site (Figure 3B).

Finally, an additional specific PCR was undertaken in order to characterize the viral genotypes circulating within our CIRDs cohort. Results showed a prevalence of the European genotypes 1A, 1B, and 4, followed by the Eurasian genotype 2B and the Asian type 2E. In particular, genotypes 1A and 1B were identified in 7 patients (1A in 4 PsA and 3 RA; 1B in 4 PsA, 2 RA, and 1 AS), type 4 in 5 patients (3 PsA and 1 RA), whereas types 2B and 2E were detected in a patient affected by PsA and in a patient with AS, respectively (data not shown).

## Discussion

Biologic therapies have successfully been introduced into the treatment of several inflammatory rheumatic diseases in particular, monoclonal antibodies or fusion proteins targeting TNF-α are widely used for the treatment of CIRDs patients refractory to conventional immune-suppressive medications. Nevertheless, treatments with biological drugs are associated with an increased susceptibility to viral infections including that by JCPyV, the etiological agent of the demyelinating disease named PML (Comar et al., 2013; Iacobaeus et al., 2013). The incidence of PML in immune-mediated diseases has recently increased as a consequence of an improved use of biologics and other potent immune-modulatory medications (Berger, 2010). Few studies are present in literature that demonstrate a real risk of PML development in CIRDs patients whereas several researches have focused on a possible correlation between JCPyV viremia and the biological therapy in patients with multiple sclerosis (MS) and CD (Lavagna et al., 2007; Verbeeck et al., 2008; Bellizzi et al., 2011, 2013a,b; Bharat et al., 2012; Comar et al., 2013; Iacobaeus et al., 2013; Tur et al., 2013; Frohman et al., 2014). Therefore, it could be interesting to understand whether there is a correlation between biologics administered for CIRDs and the opportunity that the virus escapes from latency, replicates actively and spreads to the brain causing PML. In fact, despite the limited range of species and permissive cell types for viral replication, JCPyV is a very successful pathogen because of it is able to tightly regulate its life cycle in the infected host.

In this study, 34 patients affected by RA, AS and PsA were screened to detect the presence of JCPyV in blood and in urine samples in order to evaluate the risk of JCPyV dissemination to the CNS under treatments with biologic agents and/or DMARDs and GCs. Patients were dynamically enrolled and studied from baseline, before first anti-TNF-α infusion, up to 18 months after.

A persistent JCPyV viruria significantly higher than JCPyV viremia was observed from baseline throughout the 18 month follow-up (*p* = 0.002). It could be explained taking in account that the concomitant use of conventional therapies (DMARDs and GCs) and anti-TNF-α treatments, rather than a single biologic, endorsed JCPyV replication in urinary compartment. In fact, over the time, no difference was observed between JCPyV-positive and JCPyV-negative patients treated with single administration of biologics or co-administration of DMARDs and/or GC (Table 5). Conversely, a previous study demonstrated a positive correlation between the JCPyV DNA detection in the urine and the number of biologics consecutively used for RA treatment (Verheyen et al., 2015). However, it is possible to hypothesize that the prolonged administration of biologic agents over time had a causative role in the increasing number of patients with JC viruria after 18 months of anti-TNF-α treatment. Considering that TNF-α cytokine plays an important role in host defense, it is feasible that the use of anti-TNF-α agents endorse JCV replication in the kidney. In fact, Q-PCR revealed that viral replication occurred in the urinary tract at high copy levels and that the virus could escape into the peripheral circulation as demonstrated by the fact that JCPyV DNA was detected with an increased frequency in plasma and PBMCs at T18. In addition, the effect of an ongoing immunosuppression on the JCPyV replication is confirmed by the striking number of urine positive samples detected at T18. These data allow an intriguing speculation: JCPyV might directly trigger joint inflammation. Indeed, it is well-known that viral infections can directly act on the immune system through the secretion of pro-inflammatory cytokines or favoring the production of autoantibodies (Franssila and Hedman, 2006). Therefore, urine JCPyV loads not only could be supported by inflammatory state but also could be continuous due to this insult.

**Table 5:** **JCPyV DNA in urine of CIRD patients during the 18 month follow-up**.

**Features**	T0^*^			**T3**			**T6**			**T12**			**T18**		
	**JC viruria (+) ^a^*n* = 16**	**JC viruria (−) ^a^*n* = 18**	***p*-value^e^**	**JC viruria (+) ^a^*n* = 13**	**JC viruria (−) ^a^*n* = 20**	***p*-value^e^**	**JC viruria (+) ^a^*n* = 14**	**JC viruria (−) ^a^*n* = 17**	***p*-value^e^**	**JCV viruria (+) ^a^*n* = 13**	**JC viruria (−) ^a^*n* = 18**	***p*-value^e^**	**JC viruria (+) ^a^*n* = 25**	**JC viruria (−) ^a^*n* = 7**	***p*-value^e^**
Age (range)	54.5 (36–74)	48 (25–70)	0.067	56 (32–71)	48.5 (25–74)	0.293	54 (36–74)	48 (25–70)	0.112	53(38–74)	48 (25–70)	0.085	49 (32–74)	58 (25–66)	0.386
F/M (NA)	9/7	13/5		8/5	13/7 (1)		8/6	12/5 (3)		9/4	12/6 (3)		14/11	0/7 (2)	
BA^b^	*n* = 5	*n* = 4	0.552	*n* = 5	*n* = 11	0.353	*n* = 4	*n* = 8	0.293	*n* = 6	*n* = 10	0.605	*n* = 12	*n* = 2	0.360
BA^b^+ DMARDs^c^	*n* = 4	*n* = 5	0.855	*n* = 3	*n* = 5	0.900	*n* = 3	*n* = 5	0.330	*n* = 4	*n* = 4	0.592	*n* = 3	*n* = 3	0.065
BA^b^+ GCs^d^	*n* = 5	*n* = 4	0.552	*n* = 3	*n* = 2	0.306	*n* = 3	*n* = 1	0.199	*n* = 2	*n* = 2	0.726	*n* = 6	*n* = 2	0.805
BA^b^+ DMARDs^c^+ GCs^d^	*n* = 2	*n* = 5	0.272	*n* = 2	*n* = 2	0.643	*n* = 4	*n* = 3	0.469	*n* = 1	*n* = 2	0.751	*n* = 4	*n* = 0	0.258

* *Biological agents were not yet administered at the time of the enrollment (T0). At this time point patients were treated or not with conventional therapy (DMARDs and/or GCs)*.

a *n, number of patients*.

b BA, biological agent

c *DMARDs, disease-modifying anti-rheumatic drugs*.

d *GCs, synthetic glucocorticoids*.

e *p < 0.05 was considered statistically significant*.

The hypothesis of a role of biologics in promoting viruria is also sustained by the results of an our previous study, in which we observed a significantly increased JC viruria in young patients with Crohn's disease treated with infliximab respect to those receiving a standard therapy (Bellizzi et al., 2011). Regarding viremia, it was mainly associated to viruria during the entire follow-up (Figure 1). Although JC viremia seems to be essential for the development of PML and it was detected in 37.5% (12/32) of our patients at T18, its short temporality precludes its usefulness in screening or diagnostic algorithms, as already demonstrated by other Authors in comparable studies (Rinaldi et al., 2010; Bellizzi et al., 2015; Verheyen et al., 2015). Hence, monitoring urine JCPyV replication is a good, non-invasive method to check viral pathogenic potential. In conclusion, results evidenced how viral replication and spreading could be cumulatively influenced by the use of various immune-suppressants, including biologics, rather than by a specific medications, also considering their sequential administration in the treatment refractory patients. However, due to the low number of patients analyzed in this study, how cumulative effect of various immunosuppressive agents really influence JCPyV pathogenesis, need further validation. Therefore, the cohort of studied subjects is being expanded.

In JCPyV DNA positive patients, NCCR sequencing always revealed the presence of archetype-like structures, according to other Authors (Giannecchini et al., 2012; Verheyen et al., 2015), except for a rearranged NCCR form detected in the plasma sample of a patient affected by PsA (Figure 3B). Interestingly, this type II-R rearrangement resembles the viral variants isolated in subjects who developed PML, and it is characterized by a marked neurotropism (Agostini et al., 2001; Marzocchetti et al., 2007). Indeed, it showed several repeats of the box C containing the CRE element, a specific enhancer of JCPyV replication in glial cells (Kumar et al., 1996). Moreover, it is noteworthy that this NCCR structure presents a high-affinity binding site for the specific hematopoietic transcriptional factor Spi-B. In fact, it has been demonstrated that Spi-B protein actively binds its site present on Mad-1, but not in the non-pathogenic II-S (CY) (Marshall et al., 2010, 2012). Therefore, it is possible to hypothesize that this neurovirulent variant could enter PBMCs, using them as a carrier to disseminate in the bloodstream and to reach the brain (Kumar et al., 1996). Furthermore, it is well-known that detecting viral genome in cerebrospinal fluid lied on PML diagnosis, however, its failure does not rule out the possibility that a patient might have PML, particularly in the earlier stages (Mischitelli et al., 2013). Hence, identifying in the blood a virus with PML-associated NCCR rearrangements should alert clinicians, favoring an individual management of the patient.

Finally, regarding JCPyV genotyping, VP1 sequencing evidenced that genotype 1A, 1B, and 4 were the most prevalent, although genotypes 2B and 2E were also found. Type 1 and type 4 are generally associated with Europeans and European-Americans, whereas type 2B and 2E were typical of Asians and Eurasians and of Western Pacific populations, respectively (Agostini et al., 2001). Interestingly, JCPyV subtype 2B found in PsA patient with the PML-associated NCCR rearrangement in the blood, has been associated with increased incidence of PML, while type 4 has been associated with lower disease risk (Agostini et al., 2001). Moreover, type 1 and type 4 were found in urine of Italian patients affected by immune-mediated diseases, suggesting a possible JCPyV genotype selection in response to pressure by immunomodulatory drugs (Zanotta et al., 2013).

Recently, observations of point mutations in the VP1 capsid gene have also been shown to be associated with PML (Delbue et al., 2009; Reid et al., 2011). Although VP1 gene is highly polymorphic, mutations appear to be strongly patient-related and they have been observed in virus characterized by rearranged NCCR. Practically, their arising is only noted in a neuro aggressive viral variant evolved by the non-pathogenic form, according to cell alterations or global environment changes. In summary, either VP1 mutations and NCCR rearrangements are the most common viral alterations associated with PML (Delbue et al., 2009; Reid et al., 2011).

In conclusion, since biological therapies are promising for the treatment of immune-mediated disorders, little is known about their contribution to the development of PML. However, it is clear that PML has been identified as a serious adverse event, hence it is interesting to clarify how anti TNF-α agents act on JCPyV immune-surveillance endorsing viral reactivation and dissemination. To date, epidemiology of PML has been poorly characterized among patients with rheumatic diseases due to little population-based data existing. Therefore, this study contributes to enrich literature insight on JCPyV biology in this cluster of patients, considering that the involvement of JC virus in development of adverse events in CIRDs is probably underestimated since few studies have been done about. Thus, it is necessary to carry on investigations in order to understand the real impact of biologic and/or other immunosuppressive therapies on JCPyV life cycle and to identify possible and specific viral variants that, in CIRDs patients, could be related to increased virulence.

## Author contributions

DMR, EA, MM, AB, RS, and VP designed research. DMR, EA, MM, AB, DS, GC, and CP performed experiments. All authors analyzed data. DMR, EA, MM, AB, and VP wrote the paper with contribution of ATP and MT during revision.

### Conflict of interest statement

The authors declare that the research was conducted in the absence of any commercial or financial relationships that could be construed as a potential conflict of interest.

## Footnotes

^1^ClustalW2 - multiple sequence alignment http://www.clustal.org/clustal2
